# The Oxytocin–Vasopressin Pathway in the Context of Love and Fear

**DOI:** 10.3389/fendo.2017.00356

**Published:** 2017-12-22

**Authors:** C. Sue Carter

**Affiliations:** ^1^Kinsey Institute and Department of Biology, Indiana University, Bloomington, IN, United States

**Keywords:** oxytocin, vasopressin, oxytocin receptor, vasopressin receptor subtype 1a, love, attachment, prairie voles, aggression

## Abstract

Vasopressin (VP) and oxytocin (OT) are distinct molecules; these peptides and their receptors [OT receptor (OTR) and V1a receptor (V1aR)] also are evolved components of an integrated and adaptive system, here described as the OT–VP pathway. The more ancient peptide, VP, and the V1aRs support individual survival and play a role in defensive behaviors, including mobilization and aggression. OT and OTRs have been associated with positive social behaviors and may function as a biological metaphor for social attachment or “love.” However, complex behavioral functions, including selective sexual behaviors, social bonds, and parenting require combined activities of OT and VP. The behavioral effects of OT and VP vary depending on perceived emotional context and the history of the individual. Paradoxical or contextual actions of OT also may reflect differential interactions with the OTR and V1aR. Adding to the complexity of this pathway is the fact that OT and VP receptors are variable, across species, individuals, and brain region, and these receptors are capable of being epigenetically tuned. This variation may help to explain experience-related individual and sex differences in behaviors that are regulated by these peptides, including the capacity to form social attachments and the emotional consequences of these attachments.

There is no fear in love: but perfect love casteth out fear. (1 John 4: 18)

## Introduction

Oxytocin (OT) and vasopressin (VP) are ancient peptide molecules with many behavioral and physiological functions. These pleotropic peptides evolved from a single genetic source (1). OT and VP, with their receptors, function as an integrated, adaptive system, allowing the mammalian body to survive, maintain homeostasis, and reproduce in an ever-changing world. However, OT- and VP-like molecules were co-opted for other functions many times over the course of evolution (2).

**Figure 1:**
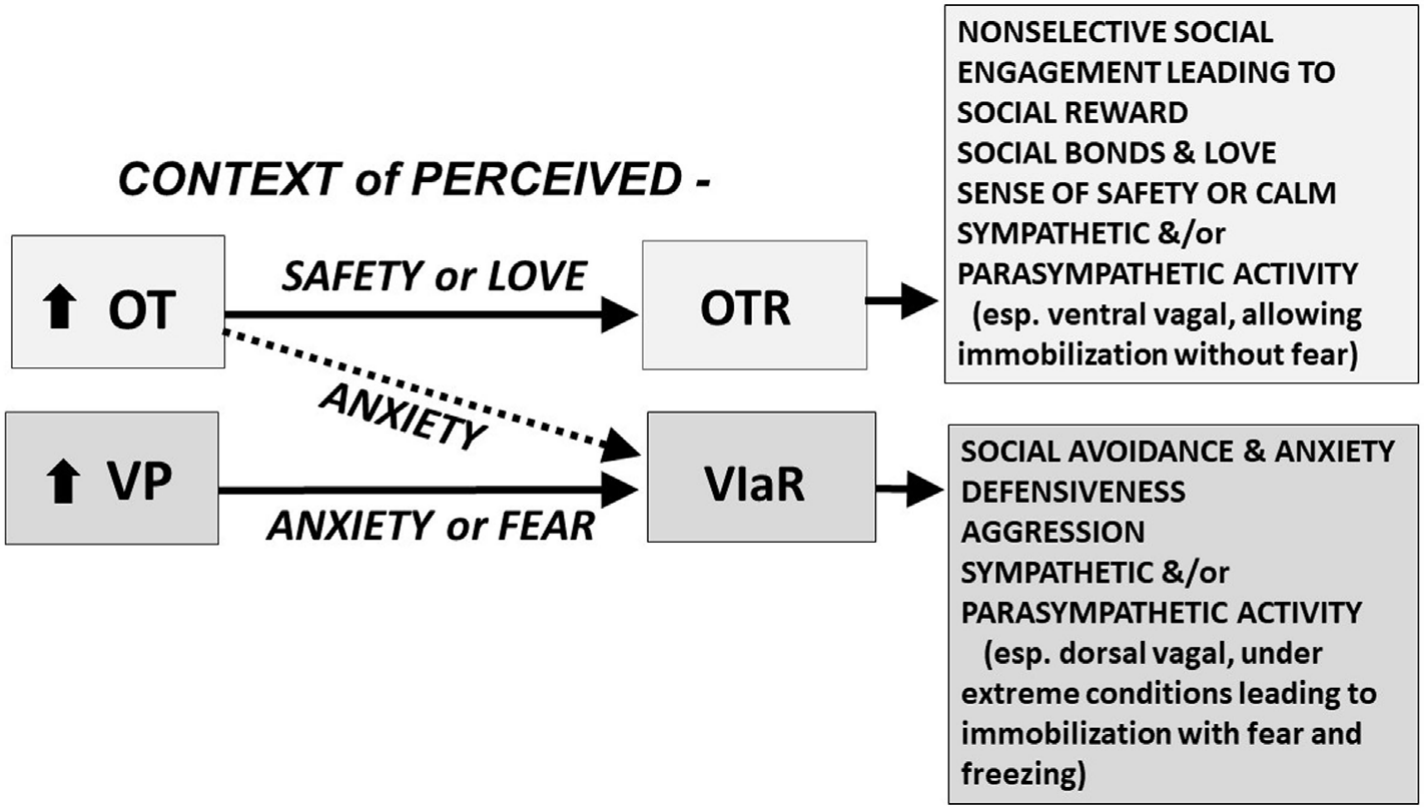
The oxytocin (OT) and vasopressin (VP) pathway includes the OT receptor (OTR) and the V1a receptor (V1aR). We hypothesize that in a context of perceived safety, OT predominately acts on the OTR, facilitating “immobility without fear,” including high levels of social engagement, social bonds, and social reward; these behaviors are at the heart of mammalian reproduction and “love.” VP and the V1aR are more ancient and probably become dominant under conditions of anxiety or trauma. In a context of anxiety or fear, OT may function primarily through effects on the V1aR; under these conditions both OT and VP may act, *via* the V1aR, to induce additional anxiety, social avoidance, defensiveness, aggression, and fear. We hypothesize that under extreme conditions, fear and the V1aR may dominate leaving the individual vulnerable to “immobility with fear,” which may lead to freezing and cognitive and emotional dissociation. These responses are mediated in part by interactive effects of OT and VP on the sympathetic nervous system and the parasympathetic nervous system, including the ventral vagal complex (necessary for social engagement) and the dorsal vagal complex (functioning to conserve energy and protect against shutting down in the face of trauma) (5). Other components of this adaptive system including the V1bR, and many other molecules or receptors, including those regulated by CRH, dopamine, opioids, GABA, and serotonin, play a role in the expression of social and defensive behaviors. The differential actions of OT and VP are dose, time, and brain-region dependent. The OT and V1a receptors are affected by genetics and epigenetic tuning, especially in early life.

Vasopressin is considered the more ancient molecule, with a central role in defense. OT, especially in a context of safety, may override the defensive functions of VP helping to facilitate the evolution of the complex cognition and selective sociality associated with human behavior, including social attachment and love (3, 4) (Figure 1).

Sources of individual differences in OT and VP and the sensitivity of their receptors include gender and basic genetic differences (6, 7). For example, some species, including humans and other socially monogamous mammals, such as prairie voles and dogs, have high levels of OT (8, 9) and an apparent dependence on OT to allow the expression of high sociality and attention to positive social cues. The OT receptor (OTR) and V1a receptor (V1aR) also can be epigenetically tuned by experience (10–14), increasing the capacity of OT and VP to have complex adaptive functions.

Behavioral work in this field has focused on the neurobiology of OT in social behavior and the management of stressful experiences (3, 4, 15, 16). The systems necessary for actions of OT involve extensive neural networks through the brain and autonomic nervous system. Many recent reviews describe the neural and behavioral roles of these peptides (4, 17–25). Furthermore, these networks are capable of dynamically changing (20, 26, 27), especially in early life (26, 27). Those reviews will not be duplicated here, but in conjunction with primary sources are used as background for a discussion of functional interactions between OT and VP and their receptors in the context of evolution and mammalian social behavior.

### The OT and VP Pathway

**Figure 2:**
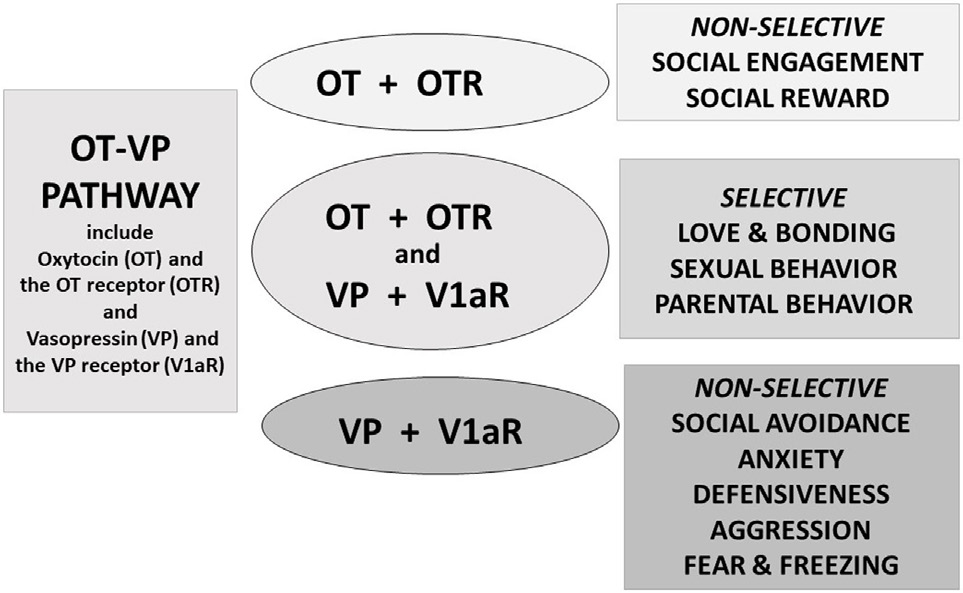
Oxytocin (OT) and vasopressin (VP) are components of an integrated pathway. OT and VP interact dynamically with receptors [including the OT receptor (OTR) or V1a receptor (V1aR)] to influence social engagement and defensive behaviors. In many cases, OT acts in conjunction with VP, *via* the V1aR or through effects on both the OTR and V1aR, thus regulating the capacity to form selective social behaviors. OT rarely acts alone but, especially under nonthreatening or “safe” conditions, may facilitate features of “love,” including social engagement, and social reward, and “immobility without fear” (36).

Current knowledge concerning OT and VP and their receptors indicate that these are interactive components of an evolved and integrated system—here termed the OT–VP pathway (Figure 2). It has long been known that both peptides can bind to both the OT and VP receptors *in vitro* (28–32). Accumulating evidence dealing with diverse outcomes and from various species supports the hypothesis that when looking at the whole organism OT and VP tend to affect more than one receptor and several types of behavioral functions (7, 20, 33–35). In general in the behavioral literature, OT has received more attention than VP.

The OT–VP pathway allows the body to adapt to highly emotional situations and develop selective attachments. Such experiences require the presence of both peptides (37), as well as molecules associated with reinforcement, such as dopamine (38–40). Conditions under which *both* OT and VP are necessary for normal behavior include selective social behaviors and emotionally intense experiences, such as sexual behavior, parental behavior, and pair bond formation, as well as regulation of the autonomic nervous system (18, 41, 42).

Until recently OT and VP, and their receptors, were typically treated as independent systems. This is especially true in human studies of the effects of exogenous hormones (43–45). For a notable exception see studies by Rilling and associates, in which both peptides are being studied (46, 47).

### Properties of OT and VP

Oxytocin and VP are small peptides that are similar in structure. Both consist of nine amino acids in a six amino acid ring, formed by cysteine bonds, and a three amino acid tail with a terminal amine group. The precursors for OT and VP consist of 12 amino acids and are synthesized and released in conjunction with carrier proteins (neurophysin 1 and 2, respectively). The precursors are later cleaved into the “mature” forms of these peptides. It is also possible that precursors and fragments of OT and VP have unidentified functions (29, 48); although not well studied, it is likely that these forms and the binding of OT and VP in blood and other tissues play a role in the functional interactions of OT and VP (49).

Oxytocin and AVP are primarily synthesized in brain regions that are critical to behavioral and physiological homeostasis. Different cells in specific brain regions produce these two peptides, including the supraoptic nucleus (SON) and paraventricular nucleus (PVN) of the hypothalamus (20). Anatomical studies in rodents indicate that OT and VP are synthesized in discrete areas and in separate cells within the PVN and SON; these cells also produce a network of neural projections reaching throughout the brain and spinal cord (50). For additional details of specific neural targets for OT and VP, see reviews such as those from Wang and his associates (39, 51).

**Figure 3:**
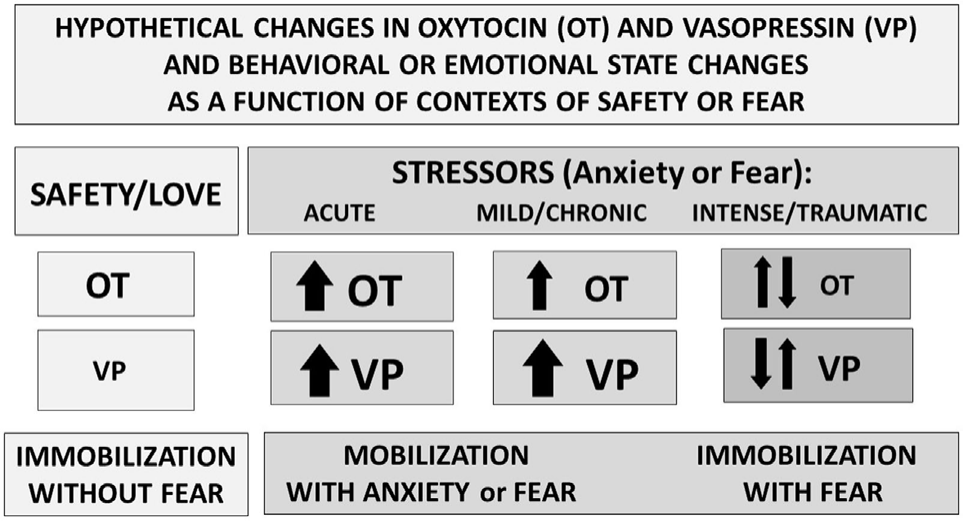
Perceived context and the intensity of challenge can regulate the release or effects of oxytocin (OT) and vasopressin (VP). Under conditions of safety, the actions of OT may dominate, supporting high levels of sociality. In response to an acute stressor, both OT and VP increase, supporting mobilization and escape, followed in some cases by increases in social behavior especially toward “safe” conspecifics. Following intense or traumatic stressors, initial responses would include mobilization and anxiety. However, following a traumatic experience, individuals may vacillate between mobilization and immobilization with fear or revert to the more primitive response of shutting down. These patterns differ between males and females and as a function of individual life histories.

Research using brain slices (25) indicates that in other brain regions, including the amygdala and the bed nucleus of the stria terminalis, both OT and VP containing cells and projections lie adjacent to each other. These OT–VP associations form local functional units, capable of rapid and often opposite interactions—for example, in brain regions associated with fear versus fear reduction. Fear responses are mediated by V1aRs in the amygdala, while OT may act to inhibit fear, depending on context (Figure 3) and gender (52).

Oxytocin and VP are synthesized and stored in the pituitary gland, where these peptides are thought to remain in vesicles until released as the nine amino acid forms. However, these molecules also may be released from axons within the CNS (20), as well as from the neuronal soma and dendrites or by diffusion within the brain (50). In addition, OT and VP are made throughout the body with local effects on diverse tissues, including the uterus, testes, digestive system, kidney, and thymus (53, 54). The dynamic nature of the OT–VP pathway not only makes this system exciting but also limits research in this field.

Sex differences are adaptive and commonly seen in studies of actions of OT, and especially VP. Sex differences are not always explored, but when they are, males and females frequently differ (52); this is especially true with reference to reactions to treatments involving stressors (3). Most of these studies suggest that males have either more VP (55) or are more sensitive to the effects of VP (56). For example, in a quantitative study of 22 subregions in the forebrain “social behavioral neural network” in rats, VP immunoreactivity show marked regional variation between males and females and as a function of age (57). These differences were particularly apparent in the medial amygdala, bed nucleus of the stria terminalis, and lateral septum—brain regions previously implicated in androgen-dependent sex differences and in defensive aggression. OT immunoreactivity did not show this pattern of variation in rats.

### Receptors for OT and VP

The gene (*OXTR*) for the OTR is found on human chromosome 3. The same OTR located in breast, uterus, and neural tissue also is present in many other bodily tissues. Three VP receptor subtypes are expressed in different tissues, and their genes are located on separate chromosomes. The V1aR is found in nervous system and throughout the cardiovascular system with a broad set of behavioral functions. The VP V1b receptor is not only found in the pituitary but also in brain areas with a role in the management of stress and aggression (58, 59). The VP V2 receptor is localized primarily to the kidney with a classical role in fluid balance.

The V1aR evolved from and is homologous with the vasotocin receptor (2). There is a high level of homology among the OTR and the three VP receptors, especially in the extracellular binding domain which allows OT and VP to bind to each other’s receptors (30, 32, 60). The pharmacological tools available for identifying, stimulating, or blocking receptors for OT and VP often have not been sufficiently selective to allow easy identification or manipulations of these receptors (61).

In mammals, receptors for OT and/or VP are typically abundant in areas of the nervous system that regulate social, emotional, and adaptive behaviors and reward (17). Among the regions with high levels of OTR or V1aR are various parts of the amygdala, the bed nucleus of the stria terminalis, the nucleus accumbens, brainstem source nuclei for the autonomic nervous system (25, 39, 62), and systems that regulate the hypothalamic–pituitary–adrenal (HPA) axis. OTRs also are found in cortex and hippocampus; these are highly variable among species and individuals, with possible consequences for neural and behavioral plasticity (63–66). In the cortex and in the spinal cord, both the V1aR (67) and OTR are present, allowing the possibility for interactions in processes such as social cognition and pain (68).

Expressions of the OTR and V1aR in the nervous system are highly variable even within species; for example, brain regions and individual differences are related to functional and adaptive patterns of sociality and aggression (6, 12, 24, 62, 69, 70). VP and OT and in some cases their receptors may differ between males and females, and across the lifespan (7, 39, 51, 52, 71, 72). The receptor variation characteristic of this system, and especially of VP has been associated with species and individual variation in behavior and brain function. Although sometimes overlooked in behavioral research, both peptides can have regulatory effects throughout the entire body, including the autonomic nervous system (42) and the immune system (53, 73–75) with effects on inflammation and healing.

Initially, it was assumed that only one primary receptor existed for OT (76). Genes for the *OXTR* and the three VP receptors code for separate G-protein coupled receptors, each with a seven transmembrane domain. Peptides binding to these receptors trigger subcellular cascades. The subcellular signaling pathways are not identical for these different receptors. In addition, the capacity of OT or VP to activate a given receptor subcellular signaling pathway may differ according to the concentrations of the peptides and the regional location of receptors in the nervous system (20, 25, 61). These subcellular differences may help to explain the capacity of OT and VP to have different functions in various processes, such as birth (77), social behavior (17, 34), and reactivity to stressors (13, 16, 25). Further adding to the complexity of this system is the possibility that receptors for OT and VP can form heterodimers with unknown consequences for peptide binding (20).

### Evolution and Sociality

Oxytocin and VP are genetic and biochemical siblings. Both originated from a single ancestral gene that produced vasotocin (1, 2). Vasotocin is found in reptiles and other vertebrates and can be measured in the mammalian fetus. OT and VP differ from vasotocin by one amino acid and from each other by two amino acids. It is estimated that the ancestral peptides arose over 500 million years ago, that VP evolved approximately 200 million years ago and OT approximately 100 million years ago, originally through gene duplication (1). The genes for OT and VP reside near each other on human chromosome 20, lying in opposite transcriptional orientations (48).

Compared with OT, VP is the more primitive molecule and closer in function to vasotocin (1, 2, 23). However, other OT-like peptides, including mesotocin and isotocin, have functions that resemble those of OT. Animals that evolved from reptiles, including mammals and birds may be particularly dependent on selective social behaviors and OT-like peptides for reproduction and survival.

Vasopressin-like molecules are critical to adaptation and water balance under difficult environmental conditions. VP can support sympathetic arousal, mobilization (flight–flight responses) or in more extreme cases a metabolically conservative, shutdown response (5). Thus, under conditions of extreme stress or trauma, VP may take precedent over OT and over survival strategies that are more prosocial or mobilized (Figure 3). However, the benefits of either sociality or OT also may be most easily detected in the presence of a stressor or of VP (3, 37).

### Combined Actions of OT and VP

At the core of positive social behaviors are neurobiological systems that regulate fear and threats versus safety (Figures 1–3). OT typically supports immobilization without fear, necessary in interactions with family and friendly associates (36). VP supports mobilization, and in some cases defensive aggression and protection of social boundaries. By contrast, VP, in conjunction with CRH, dopamine and many other molecules, may support active and mobilized coping strategies (3, 71, 78). However, as with many features of the OT–VP pathway, exceptions exist—possibly because of the capacity of OT and VP to interact with each other’s receptors (Figure 1).

Increasing evidence suggests that the actions of OT on the V1aR, versus the OTR, vary depending on the behavior and context being examined (7, 17) (Figure 1). In hamsters, fear-based or aggressive effects of OT rely on the V1aR and social reward on the OTR (34, 79, 80). The capacity of OT and VP to bind to each other’s receptors adds complexity to attempts to understand both peptides. However, OT–VP interactions also are adaptive, increasing the capacity for a small number of peptides and receptors to regulate various processes across different tissues.

Both OT and VP are responsive to environmental and social demands, although in somewhat different ways (3, 16). These peptides—presumably *via* interactions with their receptors—may have diverse physiological and behavioral properties. Regional effects of OT and VP are expected and need to be investigated to fully understand the functional consequences for these peptides. Dynamic interactions either on specific receptors, due to brain region-specific actions, or due to relative availability of the peptides to a receptor (20) could help to explain the behavioral properties of these two molecules. In addition, refined behavioral studies are necessary, since the effects of OT and VP on various behaviors change across time and as behavioral context changes.

As detailed below, a number of studies have attempted to separate the effects of OT versus VP on reproduction, social behavior, and aggression (7, 17). In general, it appears that OT plus VP may be especially critical to allow selective social experiences that involve awareness the individual identity of a partner and the experience of a social reward (18, 37). These behaviors also may require alternating between behavioral mobilization and immobilization, which is seen after trauma (36) (Figure 3).

### Methodological Limitations and “Cross Talk” Between OT and VP

The evolved properties of OT and VP permit “cross talk” between these peptides and their receptors (7, 17, 20, 31, 33, 77) (Figure 1). Unexpected outcomes are sometimes reported when exogenous OT or VP is given or when a peptide or specific receptor is inactivated. This work initially depended on pharmacological agonists or antagonists, often using drugs that were relatively non-specific. More recently research, primarily in rodents, has used genetic manipulations including rodents with mutations (81) or optogenetic methods for silencing or activating genes for peptides or receptors (40, 82).

Early evidence for cross talk between VP and the OTR came from research in OT knockout (OTKO) mice (31). For example, single-unit recording from tissue slices from the ventromedial hypothalamus from OTKO mice revealed that VP was capable of stimulating this brain region. Moreover, whether findings from mutant mice can be generalized to wild-type animals remains unclear. For example, OTKO mice showed increased sensitivity to VP. This important finding suggested that the absence of OT across the lifespan could sensitize animals to later VP exposure. The molecular basis of this process remains to be discovered. Components of some functions, such as birth and maternal behavior, continue to be observed in OTKO mice (83, 84). However, upon careful examination, these behaviors often lack the full range of behavioral expression typical of wild-type animals (81).

Similar methodology also has been used to study the OTR. Reductions in social behavior and cognitive flexibility and increases in aggression and seizure susceptibility are seen in OTR-null mice (85). These behaviors in OTR-deficient mice can be rescued not only by OT but also by VP treatments. This may be another expression of the capacity of the nervous system to adapt to changes in the peptidergic systems. However, studies of animals that are missing only one allele for the gene regulating the OTR show selective deficiencies in social behaviors, but not aggression (85). This study further supports the hypothesis that positive social behaviors may be especially sensitive to the reductions in OTR activity, while more defensive and perhaps more primitive processes are preserved.

Interactions among OT and VP and their receptors allow adaptive functions in time frames that are both short term and long term. Studies comparing the short-term versus long-term interactions between OT and VP are rare. However, those studies that do exist suggest that acute versus chronic actions of OT and VP can be very different (Figure 3), and sometimes opposite in function (86). Based on the behavioral patterns that are seen following acute versus chronic exposure to exogenous OT, we can hypothesize that the long-term effects of OT, and possibly the effects of very high levels of OT may involve stimulation of the V1aR (Figure 1).

Behavioral studies dealing with OT’s capacity to affect VP receptors have focused on OT’s effects on the V1aR or combined effects of OT and VP on the OTR and/or the V1aR (Figure 2). In general, the combined effects of OT plus VP are associated with highly rewarding experiences including some components of sexual behavior, parental behavior, and pair bond formation. At present, only a very limited number of studies seem to support the notion that OT functions primarily at the OTR without the participation of the V1aR (7, 82). Among the functions that seem especially dependent on OT are comparatively “modern” mammalian functions including lactation, reversal learning, and behavioral plasticity (33, 34, 61). Whether VP can stimulate the OTR *in vivo* has received less attention (80).

## Interactive Functions of OT and VP

### Caveats

Examples of specific studies of functional interactions within the OT–VP pathway are described below. In some cases, only a portion of the possible interactions only a portion has been tested. In most, but not all cases, OT has been shown to have the capacity to affect the V1aR. In cases deliberately involving a stressor, effects of OT or VP that were not otherwise detected may emerge. A possible role for the VP V1bR is beyond the scope of this review, but effects of stress and OT on the VP V1bR also are possible (81). Among the many other molecules of importance to the regulation of OT and VP are CRH (87), GABA (88), dopamine (38), and serotonin (89, 90); these molecules also play roles in the modulation of stress and coping. Brain region- and cell type-specific changes are another source of variation that is relevant to understanding how OT and VP interact. New technologies, such as optogenetics, are allowing more specificity in neural circuitry but are currently limited to comparatively simple behaviors or components of behavioral patterns. There is increasing evidence that the OT–VP receptor pathway is epigenetically tuned by experience, including gonadal hormones, stressors, and probably peptides as well (10, 11, 13, 14, 75). Although not reviewed here, processes such as methylation may be of particular relevance to explaining the role of context and experience in the regulation of social behavior.

### Lactation

Lactation is a defining feature of mammals, and contraction of breast tissue and milk ejection requires stimulation of the OTR. Lactation arose in conjunction with the evolution of mammals and is one of the comparatively few reproductive functions that do not continue in the absence of OT or the OTR (81, 83, 84). Immature mammalian offspring depend for varying periods of time after birth on their mother’s milk. Conservation of fluids is necessary for lactation and effects of VP on the kidney and blood pressure probably support normal milk production, but this is presumably under separate control from milk ejection.

### Birth and Uterine Contractions

Observations at the beginning of the twentieth century offered early evidence that OT and VP interactions are components of the normal functions of these peptides. Research conducted by Sir Henry Dale in 1906 showed that an extract from the human posterior pituitary gland was capable of producing contractions in the uterus of a pregnant cat. The pituitary gland contains both OT and VP and the effects of pituitary extracts probably reflected the effects of both peptides and possibly other hormones (91).

In his Nobel Lecture, describing the functions of the first “polypeptide,” Vincent du Vigneaud mentions two assays used to test the biological activity of OT. In that research, du Vigneaud (Nobel Lectures, 1955, p. 461) used rat uterine strips, but also noted that he used the “chicken vasopressor method of Coon, which utilizes the property of OT to lower the blood pressure of the fowl and has been adopted by the United States Pharmacopeia as the method for assay for OT.” The use of a vasopressor response to assay OT, indirectly acknowledged the capacity of OT to stimulate the VP system. This was one of the first of what would eventually be many lines of research documenting interactions between OT and VP.

In large mammals, OT adopts a central role in reproduction by helping, in some cases, to expel the big-brained baby from the uterus (4, 18). However, in mice and presumably other mammals, birth can occur without OT (81, 83, 84). Egg laying, which is the precursor to birth, appeared long before the evolution of mammals, and thus may rely on more ancient hormones, including VP or vasotocin.

Although OT has been assumed to play a fundamental role in birth, current evidence suggests that OT alone acting on the OTR is NOT capable of inducing normal labor and blocking only the OTR does NOT prevent premature birth. Rather both OT and VP and both the OTR and V1aR regulate uterine contractions (60, 92). Thus, it is not surprising that female mice made mutant for OT or the OTR remain capable of giving birth (83, 84). In fact, especially under conditions of stress, VP is likely to have a much greater role in birth than has been acknowledged. VP’s effects on the uterus, although functionally different from OT, may help to explain premature labor and preeclampsia, which are associated with adversity or stress across the life span (77, 91).

### Parental Behavior

Early research on OT revealed consequences for maternal behavior (93) and filial bonding (94). Although, a role for OT in maternal behavior is now widely accepted, this work was initially controversial (95). Apparent discrepancies regarding the necessity of OT to maternal behavior may have been due to experimental differences related to the role of stress in mothering. Effects of acute OT seem to be most apparent in the face of novelty, acute stressors or against a background of elevated HPA axis activity (96, 97). In the presence of OT, avoidance or fear of the infant may be replaced by approach and positive emotional states (3). Whether this is due to competitive inhibition of VP or more specific actions of OT on the HPA axis deserves additional study.

A functional role for VP in maternal behavior cannot be excluded. Pedersen and colleagues found that centrally administered VP increased maternal behavior in rats, although the effects of VP took longer to appear than those seen after OT. OTKO mice remain maternal to some extent, but their behavioral patterns are not identical to those in wild-type mice (81). The role of OT in maternal behavior may depend in part on the capacity of OT to directly or indirectly override the defensive effects of VP and reduce fear in the presence of young animals. VP, in conjunction with OT, also supports the capacity to protect offspring, in the form of postpartum maternal or paternal aggression in rodents (98, 99).

### Threatening Environments and Aggression

Vasopressin and the V1aR may be of critical importance in the capacity for physical and emotional adaption in the presence of stressful experiences (3, 16, 24, 25). VP is involved, synergizing with CRH (78), in hypothalamic regulation of the pituitary, supporting the release of glucocorticoids and mobilized defense strategies against various physical and emotional stressors or threats (25). OT also can be released during stressful experiences and is sometimes considered a “stress-coping” molecule.

Vasopressin also plays a protective role in the behavioral defense of self and the family (3, 100). Various forms of aggression and territoriality have been related to stimulation of the V1aR in both males and females (7, 101, 102). However, at least in golden hamsters the mediation of dominance and aggression was associated with increases in hypothalamic VP in males (but not in females). By contrast, serotonin, acting in the dorsal raphe, was associated with increased aggression in females, and decreased aggression in males (90).

### Avoidance of Danger and Anxiety

A growing literature associates increased central VP in the development of memory necessary for the avoidance of danger or survival (24). Psychological processes associated with anxiety and obsessions also may rely on VP (7, 103, 104). VP, in the context of other centrally active molecules, such as CRH, dopamine, and serotonin, regulates emotional states, including anxiety (Figure 3). Anxiety in turn can reduce the capacity to use cognitive or “top down” strategies to manage stressful experiences. VP and CRH can amplify the effects of each other on aggression and anxiety, especially during circumstances involving intense challenges (101, 103).

Increased activity in the central VP system may lower thresholds to impulsive forms of aggression, possibly by reducing cortical inhibition (105). The actions of VP also help to explain the association of anxiety and ruminations with cardiovascular risk (106). VP plays a central role in circadian rhythms and is likely to be important in sleep disturbances or elevations in blood pressure, which are also common following stress and considered defining features of posttraumatic stress (PTS) disorders. In human males, high blood levels of VP have been correlated with emotional dysregulation and aggression (107).

Vasopressin is associated with physical and emotional mobilization and helps support vigilance and behaviors needed for guarding a partner or territory (3), as well as other forms of adaptive self-defense (103). Prairie voles have provided a useful model for examining the importance of peptides in selective aggression (108). In this species, immediately after mating males became lethally aggressive toward strangers, but not familiar partners or family members; this response was blocked by antagonists for the V1aR (109). The formation of partner preferences and pair bonds requires access not only to the V1aR but also the OTR (37). Mate guarding and parental aggression offer examples, among several, suggesting the importance of both OT and VP and their receptors in behaviors that are socially selective (Figure 2).

### Non-Selective versus Selective Social Behaviors

Oxytocin’s role in social behaviors has been documented in many species, including humans (4, 18, 110, 111). Based primarily on work in nonhuman animals, in many, but not all cases, the effects of OT are mediated *via* VP receptors. This seems to be the case in behaviors that are non-selective, such as a general tendency toward sociality or gregarious behavior (Figure 2). This may include behavioral patterns involving social recognition (56, 112). Among other examples, in which both OT and VP receptors were examined, are social contact, including lying adjacent to another member of the same species (113) and huddling with conspecifics in the presence of the odor of a predator (114); these were facilitated by OT but only when the V1aR was accessible. In another example, in golden hamsters the effects of OT on social reward required access to the OTR (80). By contrast, in hamsters for OT to affect aggression, activation of the V1aR was necessary (79).

Research, initially conducted in prairie voles, demonstrated the capacity of OT to increase social contact between adults (115). This work led to studies showing a role for OT and the OTR in the formation of selective social bonds (116). However, in studies in which either the OTR or VP V1a were blocked, both OT and VP receptors were necessary for pair bond formation (37). When both OTR and the V1aR were blocked animals showed very low levels of contact behavior. In pair bond formation, OT and VP interact with motivational and reward systems and may enhance or otherwise amplify the effects of other molecules including dopamine and opioids in specific brain regions, including those that have been implicated in both maternal behavior and social bonding (38, 81, 117).

### Social Learning and Conditioning

Research on the behavioral effects of the OT–AVP system began with studies of memory, including avoidance learning (118) and social recognition (56, 112). These continue to be major topics in studies of the functions of the OT–VP pathway (24, 33). Learning of context and cues, as well social salience, may be affected by access to the OTR. There is an increasing tendency to direct attention to specific brain regions. In rodents, brain systems involved in reinforcement and reward, including the nucleus accumbens and ventral tegmental area, have high levels of both OTR and dopamine. OT-related sociality, probably in conjunction with the actions of dopamine, is reinforcing (40, 82). Only a few studies have suggested functions in which OT acts solely *via* the OTR, without access to the V1aR. For example, in mice, exogenous OT is capable of modulating fear conditioning following treatments directed at the lateral septum (119). OT in the lateral septum reduced fear following a positive social encounter but facilitated fear conditioning after a prior negative social encounter (120). In rats, fear conditioning also was enhanced by OT administered in the bed nucleus of the stria terminalis; blocking access to the OTR eliminated conditioned fear responses, while non-conditioned fear responses were not affected (121). In addition, in rats, effects of peripherally administered OT on neural activation in the central amygdala (indexed by cFos expression) continued to be present even following treatment with a V1aR antagonist. OT may act to increase sociality in the face of fear or challenge, including effects of exogenous OT measured by regional change in cFos in other brain areas. As one example, neural activation by OT in the hypothalamus and brain stem did require V1aR stimulation; among the other brain regions in which cFos was increased by OT and blocked by a V1aR antagonist were the SON, PVN, locus coeruleus, and nucleus tractus solitarius (35). The latter brain areas have many functions, including regulation of the autonomic nervous system and HPA axis, which are necessary for the optimal expression of social behavior (5).

### Dose-Dependent Effects of OT: More Is Rarely Better

When infant prairie voles received a low dose of exogenous OT immediately following birth, they showed as adults increased OT in the CNS and an increased tendency to form a pair bond. However, when higher doses of OT were administered, a single exposure to OT in early life disrupted the later capacity to pair bond. Females exposed neonatally to a high dose of OT later preferred a stranger. Stranger preference in prairie voles is very atypical (122, 123) and, especially in males, is most commonly associated with stressful experiences or stress hormones including CRH and cortisol (87, 124).

These and many other experiments suggest that the effects of OT are dose dependent. Low doses may appear to be beneficial, while higher doses of OT can have detrimental behavioral consequences and in some cases may stimulate the VP receptor. Low to moderate doses of OT, especially as acute treatments may reduce anxiety in the face of a challenge or stressor. By contrast, larger amounts of OT, especially if given as a chronical treatment may no longer be anxiolytic, and can have the opposite effect. Chronic or very high levels of OT can reduce the capacity to respond to OT possibly by reducing OTR or binding to the OTR, while also allowing OT to activate VP receptors (86). In another example, when male mice were tested in a social stress paradigm, chronic and high levels of OT (given centrally) were associated with an increase in anxiety-like behaviors; in that study OTR binding was also reduced in the amygdala and septum (125). Perhaps in individuals primed by negative experience, small amounts of OT are capable of activating VP receptors, further supporting mobilization and potentially defensive emotional or behavioral responses. Based on data from OTKO mice, in which the VP system was sensitized (31), we also can hypothesize that individuals (including humans) with low levels of endogenous OT might be more likely to experience increased VP-like activities even when given OT.

Studies of OT, and less commonly VP, using intranasal infusions have generated an increased interest in the behavioral effects of these peptides. The intent is to non-invasively deliver peptides to the brain and there is increasing evidence that this is possible (126, 127). However, it is useful to note, based on imaging studies in rodents, that the tissues activated by exogenous peripheral versus central applications of OT are not identical (128). Furthermore, the concentrations chosen for most human studies are generally arbitrary and based on doses of OT medically available as an intranasal “lactational aid.” Studies using different amounts of OT are needed to examine possible threshold differences among individuals as a function of gender, experience, and emotional lability (129). Different doses of a given peptide can produce different effects, and dose–response curves are only just appearing in this literature (130).

### Are There Unique Functions for OT?

In mammals, we have argued that under optimal conditions OT appears to serve as a physiological metaphor for “safety” (18). OT is of special relevance to physical and mental protective adaptations that involve high levels of sociality, a sense of psychological safety within a family or familiar social group, as well as emotional regulation that is necessary for mental health and higher levels of rational cognition (4). At least in rodents, OT seems to play an important role in cortical functions necessary for social cognition (24) and social reward (79).

Oxytocin also promotes autonomic flexibility in the face of threats (42). Parental care and social support in a safe context are particularly important in species of mammals adapted to live in extended families or groups, including humans and prairie voles (131). Social contact between adults or adults and offspring is a defining feature of most families. However, social contact, necessary for mating, parental behavior and nursing, can be dangerous and requires a physiological and autonomic state that permits “immobilization without fear” (36). This behavioral response may be especially adaptive in females but also may leave females more sensitive than males to the consequences of traumatic experiences and symptoms of PTS.

Mammals, with their comparatively large brains, are particularly vulnerable to the need for oxygen, and under extreme conditions the functions of OT may shift from social behavior to survival and protection of the cortex, including dissociation or even loss of consciousness (4) (Figure 3). In mice, exogenous OT elicited a transient activation of cortical regions and a sustained activation of hippocampal and forebrain regions. It is interesting to note that in mice intranasal VP produced a sustain deactivation of pathways associated with cortical function. Many effects of VP still existed when OTRs were genetically deleted, presumably reflecting the capacity of VP to activate cortico-parietal, thalamic, and mesolimbic regions *via* VP V1aRs (105). Whether V1aRs, possibly responding to OT, can assume such roles in primates needs additional study (62).

### Does OT Act Alone?

Many important functions including birth and selective social behaviors, another form of learned behavior, appear to rely on *both* OT and VP and their receptors. It is uncommon to find evidence that OT functions solely *via* the OTR. Lactation is one comparatively “modern” function of OT (81). Social reward may be another OT–OTR based function (79, 80), perhaps requiring a co-activation of localized dopaminergic systems (17).

Under circumstances of acute stress or prolonged isolation OT (in females) can be released (132) (Figure 3). If acting on the OTR or the V1aR this OT could allow stress coping (16). However, especially after early-life adversity, epigenetic sensitization or upregulation of VP (133) and V1aR (11) can occur. Under these conditions, OT may no longer be sufficient to be protective. Furthermore, OT may stimulate VP receptors. Thus, although OT is normally protective against stress, if it acts on the VP receptor system the effects may be seen as exacerbation of stress reactivity or anxiety. This may be a particular problem in individuals with a history of trauma and neglect, for whom the effects of exogenous OT have been reported as socially negative or “antisocial” (110, 134, 135).

### Mechanisms for OT–VP Interactions

The mechanisms underlying OT–VP interactions *in vivo* remain largely to be understood. In the face of a challenge, the interactive effects of OT and VP appear to be hierarchical. The more modern peptide, OT, may act *via* the presumably older V1aR either as an agonist or perhaps as a competitive antagonist (Figure 1). Furthermore, the functions of OT and VP may be regulated by various other processes, including differential availability of OT or VP (or their receptors) which may be regulated locally in the nervous system (20).

### The Paradox—Why Are the Social Effects of OT Unpredictable?

As data have accumulated, apparent inconsistencies or “paradoxical” effects of OT have emerged (44, 45). For example, a tendency toward parochial behavior and “outgroup” rejection was described in some human studies after intranasal OT (45, 134, 135). Treatment with OT also has been implicated in increased aggressive tendencies in certain kinds of computer games, an effect that was attributed to an OT-induced increase in social salience (136). These responses may be adaptive but also could reflect the kind of receptor “cross talk” described in studies in nonhuman animals.

In mice (86, 137) and voles (138), chronic OT exposure has been either relatively ineffective or even had negative effects on social behavior. When OT levels are high or chronically elevated their effects may be primarily due to stimulation of VP receptors with a concomitant downregulation of the OTR. This pattern of exposure to either exogenous or endogenous OT might support mobilization and potentially defensive responses, rather than positive sociality and a reduction in anxiety (Figure 3).

The history of the individual, including prior exposure to early-life stress, also can influence the response to OT. Early maltreatment also has been associated with an increase in endogenous OT (27, 139). In another example, individuals who described themselves are relatively lonely were less likely to show an OT-associated increase in parasympathetic activity (140). Perceived loneliness, isolation in early life or maltreatment might alter thresholds for physiological consequences of exogenous OT, also possibly by upregulating VP receptors and/or downregulating the OTR.

In the presence of a challenge or a negative environment (141), OT of either endogenous or exogenous origins, perhaps acting on VP receptors, could support arousal, including activation of CRH, the sympathetic nervous system and other components of the HPA and autonomic nervous system (5, 140). The interactive effects of OT and VP, including actions on the V1aR may help to explain the observation that treatment with OT has frequently been associated with antisocial behaviors, especially in a context of fear or danger.

## Summary

Across the lifespan, the effects of OT and VP dynamically interact to adjust to and influence the perception of fear and safety. VP is the evolutionarily older molecule with presumably the older receptors. VP is implicated in mobilized behaviors including defense of self and the family. Among the patterns of behavior for which both OT and VP may be necessary are sexual behavior (142), paternal behavior, and pair bonding (18). OT is of special relevance to adaptations that involve high levels of sociality, a sense of psychological safety within a family or familiar social group, as well as emotional regulation and higher levels of rational cognition (4). Furthermore, working together OT and VP, and their receptors, create a biological and genetic pathway that regulates attachment and bonding, which in turn may be protective against threats or other forms of challenge.

The nature of interactions of OT and VP at their receptors needs further study, especially *in vivo* and the epigenetic context of development (26). There is considerable interest in using OT-like molecules as therapeutics. However, the evolved and dynamic features of the OT–VP pathway create difficulties for attempts to study OT and VP independently. These also pose challenges for the usefulness of drugs based on this system, including those commonly used around the time of birth, such as synthetic forms of OT, which may affect both the OT and VP receptors.

## Author Contributions

CSC conceived and wrote this review.

## Conflict of Interest Statement

The author declares that the research was conducted in the absence of any commercial or financial relationships that could be construed as a potential conflict of interest.
